# Design of Twin Builder-Based Digital Twin Online Monitoring System for Crane Girders

**DOI:** 10.3390/s23229203

**Published:** 2023-11-15

**Authors:** Baogui Huang, Yanbo Hui, Yonggang Liu, Hongxiao Wang

**Affiliations:** 1 College of Mechanical and Electrical Engineering, Henan University of Technology, Zhengzhou 450001, China; 2021920272@stu.haut.edu.cn; 2 Postdoctoral Research Workstation of Weihua Group Co., Ltd., Xinxiang 453000, China; 201992296@stu.haut.edu.cn

**Keywords:** crane girder, True-Load, reduced-order modelling, Twin Builder, online monitoring

## Abstract

The crossbeam is frequently subjected to alternating loads during work as an essential load-bearing part of the crane. However, due to the large volume and the limitations of detection technology, it is impossible to realize online monitoring of the mechanical state. The ongoing advancement of ROMing and digital twin technology plays a pivotal role in facilitating the resolution of this particular issue. In this paper, we take the crane beam as the physical entity and combine the Twin Builder reduced-order technology and Deployer digital twin deployment technology to establish a digital twin of the beam. The load recognition model within the twin system exhibits a prediction error rate of ±5%. Furthermore, the accuracy of the ROM surpasses that of conventional machine learning models by a factor of 25. Upon deployment on the web platform, the results are delivered within 0.5 s, representing a substantial improvement as it is merely 1/15 of the time required for traditional 3D displays. The digital twin online monitoring system has the advantages of high accuracy and low requirements for monitoring equipment, which can be widely used in engineering practice to solve the problem that the mechanical state of large parts cannot be accurately monitored online.

## 1. Introduction

In the realm of industrial manufacturing, logistics, and related sectors, cranes assume a pivotal role as indispensable tools for driving industrial progress. Comprehensive investigations and research have unearthed a disconcerting trend wherein the operation of lifting machinery frequently results in catastrophic structural failures, particularly beam fractures [1,2,3,4,5]. These accidents can be attributed to factors such as crane overloading, microscopic metal cracks, and metal-fatigue-induced breakdowns. The recurring incidence of such grave safety mishaps underscores the imperative to maintain a vigilant focus on the structural integrity of cranes. A meticulous analysis of the accident causes reveals that many of these issues are imperceptible to the naked eye, rendering the implementation of simple protective measures challenging. The prediction of real-time stress and deformation, whether at the global or local level, in lifting machinery is critical for ensuring crane safety. Consequently, the establishment of a real-time monitoring system for tracking stress and deformation in lifting machinery plays a pivotal role in preventing crane safety accidents. The conventional approach of affixing strain gauges at fixed points for monitoring the condition of mechanical components is susceptible to the influence of structural intricacies, which hinders the acquisition of precise data. Furthermore, the sheer magnitude of crane machinery renders comprehensive monitoring unattainable. In contrast, the finite element analysis method excels in accurately forecasting the overall stress and deformation of components under specific load conditions. However, its utility is limited in real-time prediction for larger structural elements of cranes due to computational constraints. This method is better suited for addressing the requirements of smaller components. The ongoing development and enhancement of digital twin technology have equipped it to effectively address the aforementioned challenges. The pivotal factor enabling digital twins to accomplish real-time monitoring lies in the utilization of the ROM, a methodology that systematically reduces the complexity of the original model through a sequence of model simplification techniques. This results in the attainment of a low-order approximation that closely approximates the original model to a remarkable degree [6,7,8].

In recent years, both domestic and international scholars have conducted extensive research to investigate the practical application of ROMs and digital twin systems founded on these models in the field of state detection. Various techniques are employed to derive these ROMs, including Proper Orthogonal Decomposition (POD), Volterra Sequence Representation, and the Harmonic Balance method. It is noteworthy that the selection of these methods depends on the specific computational physics problem at hand, as each method exhibits distinct applicability. Saddam Hijazi et al. [9] applied the classical POD–Galerkin projection method to construct a tailored ROM designed for simulating turbulence within a finite-volume environment. Through rigorous experimentation, it was consistently demonstrated that the error between the ROM and the full-order model remained below 8%. Furthermore, this approach achieved a remarkable 90% reduction in computational time compared to the original method. D. Xiao et al. [10] integrated orthogonal decomposition techniques with advanced machine learning methods to formulate a robust, high-fidelity, and confident noninvasive ROM for monitoring ambient airflow states. This innovative model shows significant promise in supplanting the conventional Gaussian smoke plume model. David J. Lucia et al. [11] integrated the Volterra theory with tailored orthogonal decomposition techniques to formulate an illuminative model for fluid-state surveillance, yielding an error of under 5% when comparing the ROM to the full-order model. The objective behind deriving ROMs from diverse physical systems using various techniques is to lay the foundation for a digital twin system rooted in these ROMs. This digital twin system [12,13,14,15,16,17] is then deployed to oversee the condition of physical entities, evaluate their status through state monitoring data, and proactively implement timely preventive measures. Han Dong et al. [18] employed an adaptive partitioning strategy in conjunction with Galerkin methods to introduce a kinetic model designed for the rapid resolution of fractures in large components. The empirical findings demonstrate that employing this model substantially reduces the degrees of freedom while ensuring accuracy. Additionally, the computational time on the CPU is drastically reduced by almost tenfold. Furthermore, integrating this model with sensor components enables the creation of a digital twin system for monitoring the structural integrity of large components, facilitating the early detection and prediction of metal fracture risks. Khamlich et al. [19] have engineered a high-precision ROM to address the advection–diffusion challenge within environmental monitoring, employing a data-driven approach that amalgamates proper orthogonal decomposition and regression, known as POD-R. This methodology has culminated in the development of a digital twin system designed for the real-time monitoring of airflow states in correlation with environmental conditions. The system’s exceptional precision and timely data acquisition underscore its potential benefits in the realm of environmental management, particularly with respect to mitigating air pollution. Gianmarco Aversano et al. [20] have innovatively crafted a ROM of the physical system by employing a novel blend of intrinsic orthogonal decomposition and kriging interpolation. Notably, they introduce a pioneering digital twin model tailored for furnaces operating under flameless combustion conditions. This innovation empowers the accurate forecast of combustion data even when facing unfamiliar combustion scenarios. Remarkably, their digital twin consistently forecasts combustion pollutants with an impressively low margin of error, less than 5%, while simultaneously achieving an outstanding 80% reduction in computational time. This work serves as a compelling showcase of the vast potential inherent in deploying digital twins based on ROMs, particularly in the domain of condition monitoring. In the context of a jib crane, Lai Xiaonan et al. [21] have introduced a novelty approach known as ‘Shape-Performance Integrated Digital Twin’. This method is proficient in generating diverse downscaled models tailored to specific requirements. Furthermore, the team has engineered a dedicated application for visualizing 3D predictive outcomes. In tandem, they have established a comprehensive digital twin monitoring system, proficient in tracking the spatial orientation, structural stress, and velocity characteristics of physical entities through the installation of a variety of sensors. The acquired data serve as a foundation for making anticipatory assessments regarding potential operational risks. An abundance of research findings substantiates that the utilization of a reduced-order, low-order model not only upholds remarkable accuracy but also substantially curtails computational time. Furthermore, the digital twin system, founded upon this ROM, is proficient in effecting real-time monitoring of the state of physical entities.

To mitigate the likelihood of critical safety incidents within lifting machinery, this study amalgamates intrinsic orthogonal decomposition with machine learning techniques to address the stress and deformation degradation model of crane girders. It further introduces a load recognition model to furnish inputs for the degradation model, facilitating the construction of a digital twin system for real-time monitoring of the stress and deformation status of crane girders. To establish a comprehensive monitoring system, Deployer software 2021R1 is utilized. This system is integrated with strain sensors to continuously collect real-time strain data, enabling the monitoring of the crane girder’s stress and deformation status. The data are then visualized through a cloud map representing the overall stress and deformation of the beam. Additionally, a line graph pinpoints specific stress changes, facilitating an in-depth safety assessment. This approach ensures timely maintenance and overhaul of the beam, effectively averting critical safety incidents such as beam fracture and metal fatigue failure. The specific methodology is outlined as follows: initially, conducting a finite element analysis of the crane beam under defined operational conditions; subsequently, employing the True-Load software 2022R1 [22,23,24,25,26,27] for strain test pre-analysis based on the results obtained from the finite element simulations. This process includes acquiring the positional and angular details of the strain gauge layout within a carefully selected area, conducive for load inversion, and finally, utilizing real-world load and strain data to construct a load recognition model in Twin Builder. Subsequently, the ROM module within Twin Builder is employed to process the full-order finite element model. This process leverages intrinsic orthogonal decomposition and machine learning techniques to produce a ROM that aligns with the specified criteria. The result display window is then established, finalizing the construction of the monitoring system. Strain gauge data are acquired via CSV file input, and the corresponding data file input interface is preserved. An executable SDK file is generated using Deployer software, facilitating the deployment of the digital twin. To achieve real-time collection of stress–strain data and real-time data transmission interface integration in the deployment file, the API secondary development interface offered by Donghua sensor software 2021 is utilized. This culminates in the comprehensive design of the digital twin monitoring system.

## 2. Load Physical Sensing System Design

The load physical sensing system is a bridge between the physical entity and the digital twin, through the sensor to obtain the real-time strain data at the fixed point of the beam, as the input of the digital twin to achieve the interaction between the physical entity and the digital twin. The core of the system is to determine the sensing strain gauges’ patch position that can accurately invert the lifting load. For this problem, this paper uses True-Load software to invert the optimal sensing strain gauges’ patch position according to the simulation results under the actual working conditions.

### 2.1. Finite Element Simulation Analysis

**Figure 1:**
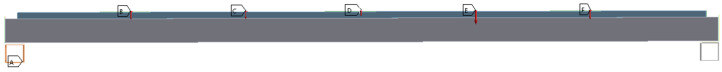
Load setting.

**Figure 2:**
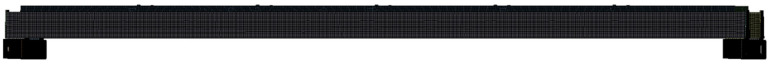
Finite element meshing.

**Figure 3:**
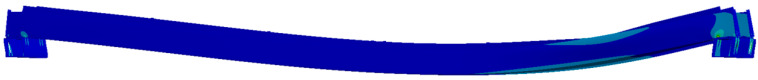
Stress map.

**Figure 4:**
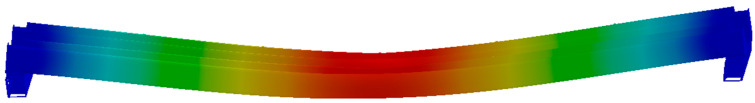
Deformation map.

The experiments are conducted on an overhead crane beam with a rated lifting weight of 1 ton, a length of 5.85 m, a width of 1.5 m, and a cross-sectional width of 20 mm as a physical entity. The beam material is Q235 structural steel with a modulus of elasticity of 206 Mpa and Poisson’s ratio of 0.3; the main body is made of square steel, and the ends are fixed by welding and bolting. Use Solid Works software 2020 to draw its equal-scale 3D model, with output as x_t general format. The model is converted into a shell unit in Space Claim of Workbench, which is convenient to extract the results for True-Load analysis. The hexahedral mesh is selected to divide the beam; the maximum cell size is 4 mm, the minimum is 1 mm, the number of mesh cells is 114,081, and the number of nodes is 111,895. When analyzing, the end weld is set up with bound contact, and the self-weight of the trolley, spreader, and crane beams is added in the way of gravity, which is 1700 N, a preload of 1000 N is given to the bolt joints, and the fixed constraints are applied to the ends of the beams. The loads were segmented into five sequential load steps and applied at positions B, C, D, E, and F, as illustrated in Figure 1. Figure 2 shows the mesh delineation of the crane crossbeam, Figure 3 shows the stress simulation result cloud, and Figure 4 shows the deformation simulation result cloud, which shows that the maximum deformation of the crossbeam is directly below the force loading point. This simulation result is used as a sample of the full-order model for the ROM, marking the loads as inputs and stresses and strain values as outputs.

### 2.2. True-Load Load Inverse Analysis

**Figure 5:**
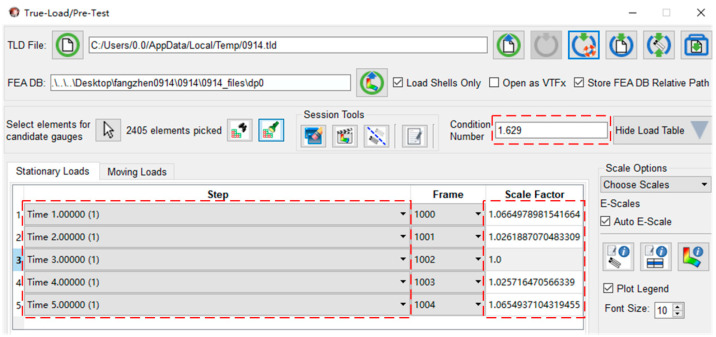
True-Load condition number results.

**Figure 6:**
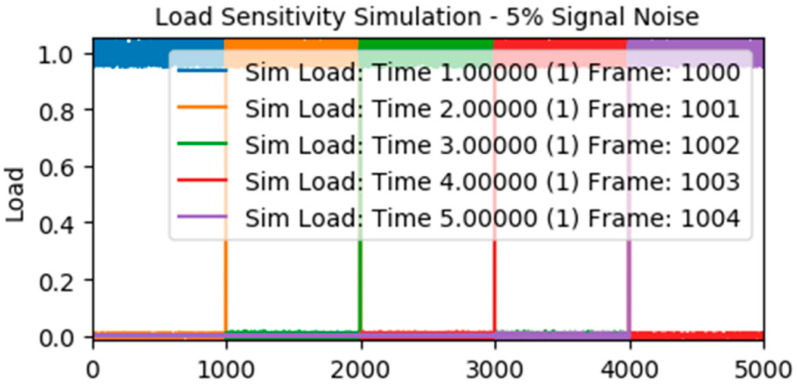
Analysis of measurement point errors.

**Figure 7:**
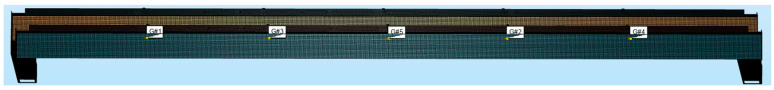
Strain gauges’ location.

Import the result file of Workbench FEA into True-Load, select the target area for the strain gauges in the appropriate position of the main beam in order to avoid too much concentration in the paste position, and break up the target area with a spacing of 10 mm. Then, the five load steps in the finite element analysis are uploaded, set as the number of strain gauges 5, and click the Start button to run the calculation to obtain the coordinates of the five best-sensed load strain gauges’ patch positions and the X, Y, Z axis deflection angles. The interface of the analysis results is shown in Figure 5. The Condition Number (CN) of the analyzed patch is 1.67, and the evaluation criteria are as follows: CN = 1 Perfect; CN = 1~10 Very Good; CN = 10~20 Good; CN = 21~50 Usable; CN > 51 Unusable. The corresponding scaling factors of the five load steps are similar, and the scaling factors are identical to each other, as shown in Figure 6, which is in line with the experimental expectation, and the patch position results are shown in Figure 7.

### 2.3. Physical Load Sensing Modelling

The function of the load sensing model is to invert the real-time lifting mass according to the values of the sensing load strain gauges pasted on the crossbeam during the crane working process, which can be realized by the built-in response surface ROM module of Twin Builder software2023R1. The steps are to take the change history of the strain gauge value as input and the change history of the corresponding lifting mass as output during multiple sets of experiments, import the response surface ROM, and generate the load sensing model, so it is necessary to carry out multiple sets of experiments to establish the control relationship between the strain gauge value and the load. When the crane is running, the trolley will move along the beam, and its position will be changed; to achieve the prediction of the mass of the lifted weight when the trolley is in a different situation, the trolley is stopped at five equal position centers. So, establish five channels to carry out multigroup lifting experiments.

**Figure 8:**
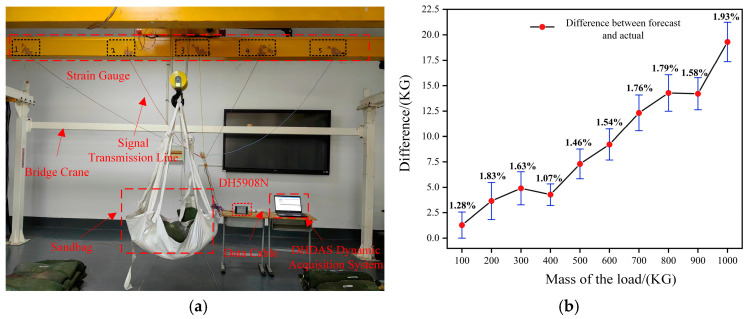
Test site and results analysis. (**a**) Strain test site; (**b**) Load error analysis.

Experiment with flood control sandbags as the weight, due to the quality of sandbags, is not uniform, according to the actual weighing situation set up: 0 N, 1014.1 N, 1977.5 N, 3048.5 N, 4121 N, 4986.5 N, 5947 N, 6890.1 N, 7796.1 N, 9025.1 N weight of the ten groups of weight lifting; 120 mm × 120 mm × 140 mm × 2 T specification soft pallet as lifting gear, Donghua test DH5908N real-time strain collector as the data acquisition device, according to the coordinates of the True-Load solution in the corresponding position of the paste strain gauges, supporting the use of the DHDAS dynamic strain acquisition system, in different positions were lifting the above different masses of weight, to be stable when the stability of the repeated recording of each strain gauge value is taken for the average of the value. The experimental field diagram is shown in Figure 8a, and the measurement results at position three are shown in Table 1. The first five columns in the table are used as the strain change history, and the last column is used as the load change history, which is imported into the response surface ROM to generate the required load recognition ROM, i.e., the physical load sensing model.

In order to verify the accuracy of the physical load sensing model, a total of ten sets of experiments were measured starting from 1000 N and spaced from 1000 N to 10,000 N, comparing the mass of the actual load with the mass output from the load recognition ROM, and the absolute value of the error rate was calculated as shown in Figure 8b. The results show that the difference between the prediction results of the physical load sensing model based on the response surface ROM and the actual load will change with the rise of the lift weight, but the absolute error rate of the two is always less than 2.0%, which indicates that the physical load sensing model has a high prediction accuracy. It can satisfy the needs of the practical scenarios.

## 3. Reduced-Order Model Construction

The ROM creation method provided by Twin Builder software carries out the intrinsic orthogonal decomposition of the finite element full-order model to obtain a set of orthogonal basis functions, which are used as the subspace for the ROM, and then construct a polynomial response surface approximation model using the data difference technique integrated with ANSYS. The ROM is obtained, which can be used for real-time mechanical analysis under different input parameters. To validate the accuracy of the ROM, the prediction model obtained from the training of the undecomposed full-order model data using a BP neural network [19,20,21,22] is used as a reference group to carry out a comparative analysis.

### 3.1. Generating a ROM

Creating a ROM requires multiple full-order models as sample data, each representing different load conditions within the effective lifting load range. The design of experiments (DOE) function provided by the Workbench software2022R1 can randomly set different input sample points according to the experimental requirements in combination with the lifting weight range. There are several methods for random sampling design, including intermediate composite design, optimal space-filling design, Box–Behnken design, and Latin hypercube sampling design. In order to ensure the practicability and extensiveness of the model sample data, this paper adopts user-defined settings. It adds 20% overload simulation experiments, setting up a total of 24 groups of sample experiments from 500 N to 12,000 N loads, with a gradient of 500 N, and saving the simulation results corresponding to the different load conditions as two folders of Stress and Deformation as the downscaled model. At the same time, the simulation results were saved in a separate data format as the training dataset for the full-order model of the BP neural network in the reference group.

**Figure 9:**
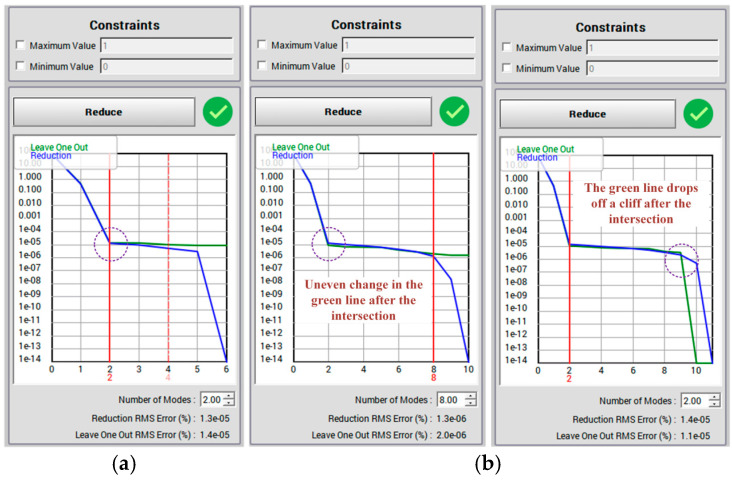
Error curve setting: (**a**) correct form; (**b**) wrong form.

The generated Stress and Deformation results folder is imported into the Static ROM Builder module in Twin Builder, and a certain proportion of data is selected as the Snapshot matrix to generate the model order error analysis curve with the descending model order as the horizontal coordinate and the error as the vertical coordinate. The blue curve is the size of the error between the ROM and the full-order model for the same point, and the green curve is the trend of the blue curve with the change in the order of the ROM. The generated curve needs to meet the following two conditions: first, after the intersection of the blue curve and the green curve, the green curve needs to be on top of the blue curve; second, after the intersection of the green curve, the trend of the green curve changes should be smooth, and there is no cliff change; this is achieved by constantly adjusting the ratio of the data. The correct case is shown in Figure 9a, and the error is shown in Figure 9b. The highest order of the ROM that conforms to the model analysis curve is significantly reduced, and the operation amount is suddenly reduced. The machine learning method is used to learn the sample data of the ROM, and the twin models that can predict the inputs of the unknown load, the stress ROM and the deformation ROM, can be obtained.

**Figure 10:**
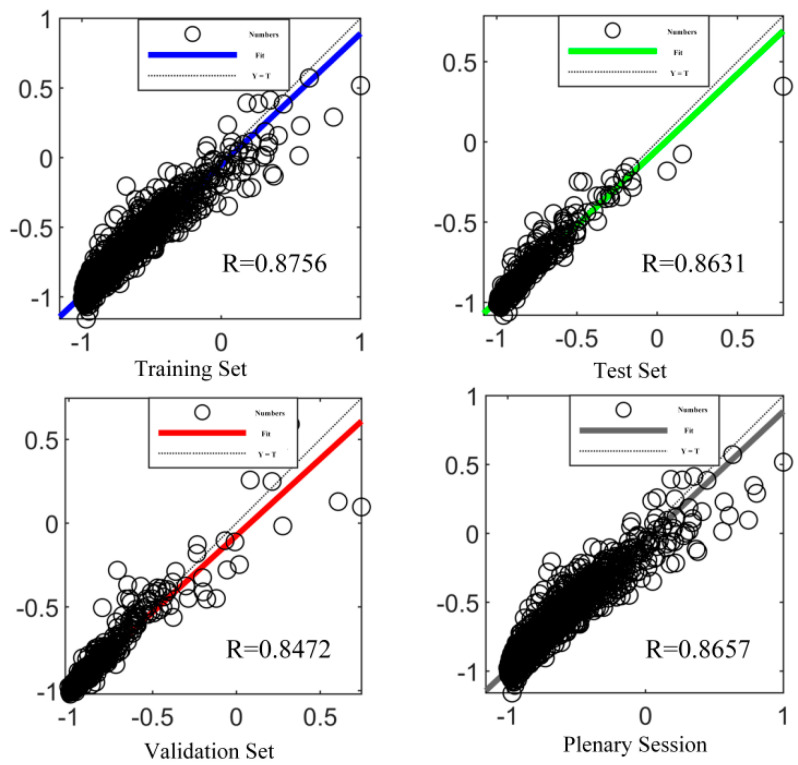
BP neural network training results.

The reference group is the extracted data imported into MATLABR2023a software in the format according to the load, node X, Y, and Z coordinates, and mechanical state data, and the training data are learned using the constructed single-hidden-layer BP neural network oriented to the Delta rule. After continuous attempts, when the number of hidden layers is 7, the proportion of training data is 80%, the proportion of validation data is 10%, and the proportion of test data is 10%, the model training effect is the best, as shown in Figure 10.

**Figure 11:**
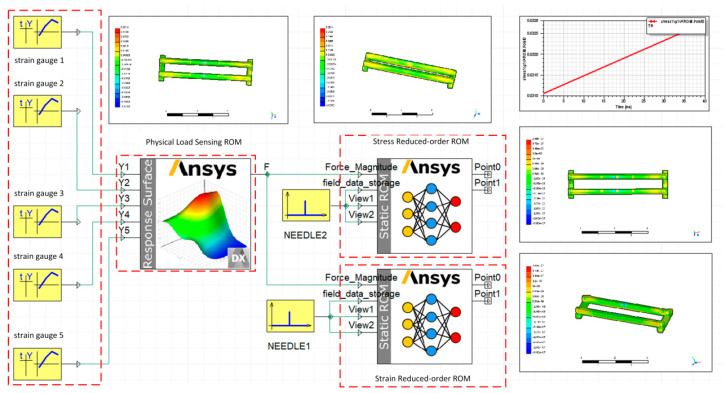
Digital twin simulation system for a single location.

**Figure 12:**
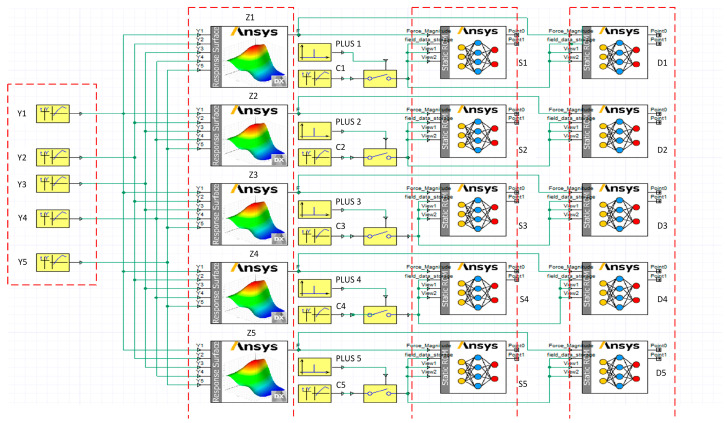
The entire digital twin simulation system.

The load recognition model and the step-down model are visualized using Twin Builder software2022 individually. Additionally, the output ‘F’ ports of the load recognition model are interconnected with the ‘Force_Magnitude’ ports of the two step-down models. This configuration facilitates the utilization of the load recognition model to furnish load information to the step-down models. Furthermore, the system configuration includes the creation of an overall stress and deformation cloud map for the beam, along with stress or deformation line graphs at specific points. This comprehensive simulation system is presented in Figure 11. Figure 11 depicts the simulation system, with the cart positioned at beam marking location 3. Similar simulation systems have been constructed for the remaining four positions following the same operational procedure. This results in a comprehensive simulation system comprising five positions, as illustrated in Figure 12. The simulation system operates by assessing the magnitudes of five strain gauge values and assigning a value of 2 to the location with the highest strain measurement while assigning a value of 1 to the remaining positions. These values are then stored in a table denoted as ‘C’. Data in ‘C’ are compared with a default value of 1 within the switch function. When it exceeds 1, the switch is engaged to execute the simulation at this position, and vice versa. In this context, ‘Y’ denotes the strain gauge numerical interface, ‘Z’ signifies the load recognition model for each position, ‘S’ represents the stress (stress) reduction model for each location, ‘D’ stands for the deformation (deformation) reduction model for the specific position, and ‘plus’ is employed to configure the pulse function, determining the frequency of the cloud output.

### 3.2. Reduced-Order Model Error Analysis

**Figure 13:**
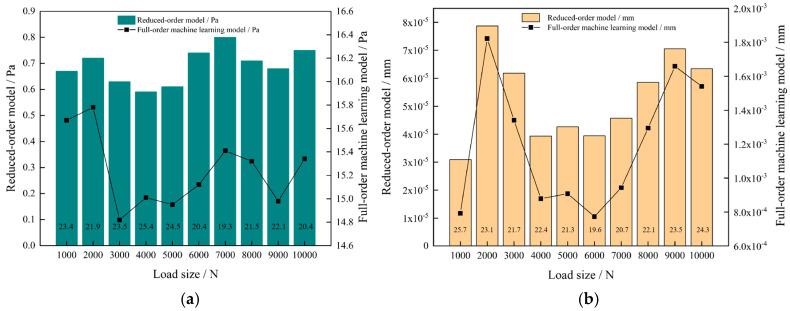
Error analysis for ROM: (**a**) Comparative analysis of stress errors; (**b**) Comparative analysis of deformation errors.

The maximum difference between the stress and deformation prediction results of the down-order model and the full-order BP neural network learning model and the finite element simulation results are calculated, respectively, under the same loading conditions as shown in Figure 13. The error between the stress and deformation prediction results of the ROM and the simulation results is minimal, which is one-twenty-fifth of that of the full-order BP neural network learning model. Meanwhile, due to the excessive amount of data processed by the full-order BP neural network learning model, the time used is much higher than that of ROM, and the comparison data are shown in Table 2. The results reveal that the BP neural network necessitates 200 times more time for computation than the ROM. Furthermore, the aforementioned findings underscore the remarkable accuracy of the ROM achieved within a significantly shorter time frame.

## 4. Deployment and Application of Digital Twin Models

The twin that meets the accuracy requirements is compiled in the Twin Builder environment to generate a twin format file, reserving the input interface for the sensed load strain gauges and the output interface for the cloud diagrams and stress change curves that need to be displayed. Then, in the Deployer software, drag in the CSV-input data input module, import the CSV file of the measured strain gauge data change history, and display each strain gauge data output interface; drag in the Twin Model module that can encapsulate the twins, import the generated digital twins of the twin format, and display the reserved input and output interfaces; and set the strain gauge data output interfaces. Connect the strain gauge data output interface with the twin one-to-one data input interface, and the twin data output interface does not need to be connected. Use the system default settings to output the SDK deployment file. Deployer provides three forms of deployable SDK file generation methods: Linux system web deployment, windows system web deployment, and a small black window deployment. After experimental comparison, web deployment can not only obtain the current moment to stress cloud map, it can also be set up through the button to call out the specific point of the stress change curve, a more intuitive image.

**Figure 14:**
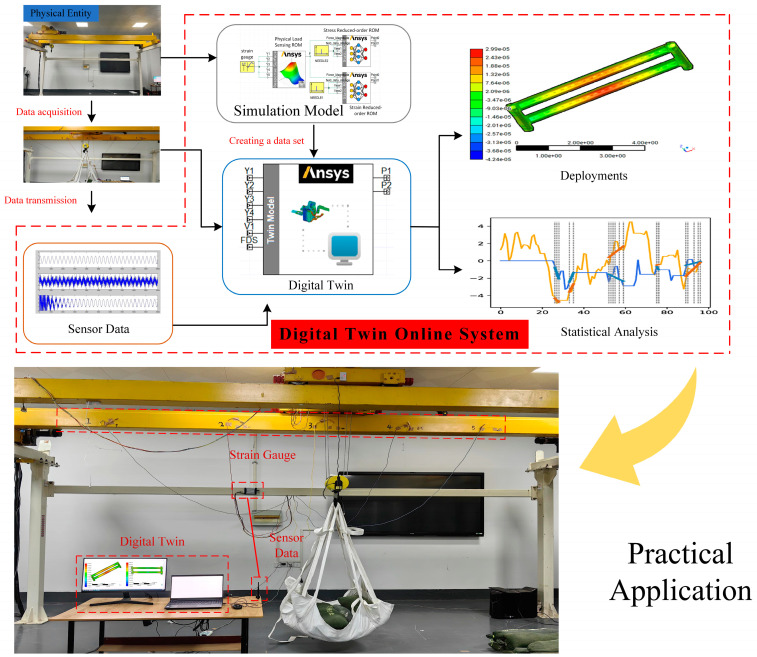
Applications of digital twins.

The above digital twin system uses the historical data collected by the sensed load strain gauges, and the real-time strain gauge measurement data still need to complete the connection with the twin system. To address this problem, this paper writes a program through the API secondary development interface provided by Donghua test software to connect the real-time collected sensed load strain gauge channel of the sensor with the strain data interface in the generated SDK deployment file and to judge the five values, and give the maximum value channel outputs the value of size for the role of the switching controller, to judge whether to output the corresponding position of the ROM and to achieve the digital twin. The deployment of the system application is shown in Figure 14.

## 5. Conclusions

To mitigate the risk of safety incidents such as beam breakage during crane operations, this study introduces a digital twin condition monitoring system centered on the reduced-order model of beams. This system is tasked with continuous real-time monitoring of both global and local stress and deformation levels within the beam. It then transmits the collected data to equipment maintenance engineers, enabling them to evaluate the safety condition of the beam. When engineers detect stress or displacement deviations in the beams beyond permissible limits, they promptly initiate maintenance at the relevant beam locations to ensure their continued normal operation. Upon comparing the output results of the ROM with those of the full-order finite element model under identical input loads, it is evident that the maximum error between the ROM employed in this study and the simulation results from the full-order finite element model is well below 1.0 × 10^−15^. This compellingly illustrates the exceptional fidelity of the ROM. The ROM’s computational time is merely 1/200 of the finite element analysis time under identical input conditions, unequivocally affirming its exceptional computational efficiency. Utilizing the Deployer software, we compile the entire monitoring system into an executable SDK file, enabling independent execution apart from the Twin Builder software. This approach minimizes the computational demands on the host system, facilitating a seamless connection to the DHDAS strain monitoring system via the data interface for real-time monitoring. The digital twin-based online beam monitoring system presented in this study holds significant engineering applicability. It not only enables swift data acquisition but also offers the advantage of minimizing computer hardware demands, thereby conserving valuable time and resources. To enhance its capabilities, this monitoring framework will be extended through the incorporation of diverse sensor types. Furthermore, the historical data amassed will be meticulously collated and subjected to rigorous analysis for predictive insights into beam life span.

## Figures and Tables

**Table 1:** Strain and Load Measurements.

Strain Gauge 1/με	Strain Gauge 2/με	Strain Gauge 3/με	Strain Gauge 4/με	Strain Gauge 5/με	Mass of Weight/kg
1.1	3.7	4.3	1.3	1.033	40.01
2.4	6.4667	7.6333	2.7333	2.3333	70.21
5.1333	13.7	15.2333	3.7333	4.59	119.71
7.1	19.1333	23.3333	8.8667	7.3333	192.69
12.0333	26.5	32.4667	12.8333	10.633	277.19
13.8667	31.5667	39.4667	15.4667	12.111	333.94
16.2	36.0667	45.8667	18.2667	15.05	393.14
17.5	41.5	52.633	19.8	16.89	452.8
20.833	46.8	59.333	23.5333	19.4	513.55
22.4667	50.8	63.9	26.533	21.1833	550.75
24.2	54.6	69.433	27.133	23.333	601.75
25.2	57.3	73.133	27.9333	24	632.4

**Table 2:** Comparison of the time of use of the two methods.

Loads/N	ROM/s	Back-Propagation (BP) Neural Network/s			Multiple of Both
		Computation Time/s	Cloud Map Display Time/s	Total Time/s	
1000	0.152	1.5	35	36.5	240.2
2000	0.149	1.4	35	36.4	244.3
3000	0.155	1.5	35	36.5	235.5
4000	0.146	1.5	35	36.4	249.3
5000	0.151	1.5	35	36.5	241.8
6000	0.148	1.5	35	36.5	246.6
7000	0.153	1.5	35	36.5	238.6
8000	0.149	1.4	35	36.4	244.3
9000	0.148	1.4	35	36.4	245.9
10,000	0.152	1.5	35	36.5	240.1

## Data Availability

Data are contained within the article.
